# Effect of myrrh and thyme on *Trichinella spiralis* enteral and parenteral phases with inducible nitric oxide expression in mice

**DOI:** 10.1590/0074-02760150295

**Published:** 2015-12

**Authors:** Rasha AH Attia, Abeer E Mahmoud, Haiam Mohammed Mahmoud Farrag, Rania Makboul, Mona Embarek Mohamed, Zedan Ibraheim

**Affiliations:** 1 Assiut University, Faculty of Medicine, Department of Parasitology, Assiut, Egypt; 2 Assiut University, Faculty of Medicine, Department of Pathology, Assiut, Egypt; 3 Assiut University, Faculty of Medicine, Department of Microbiology and Immunology, Assiut, Egypt; 4 Assiut University, Faculty of Pharmacy, Department of Pharmacognosy, Assiut, Egypt

**Keywords:** Trichinella spiralis, albendazole - thyme, myrrh, iNOS, enteral, parenteral phases, cytotoxicity tests, limulus amoebocyte lysate assay

## Abstract

Trichinellosis is a serious disease with no satisfactory treatment. We aimed to assess the effect of myrrh (*Commiphora molmol*) and, for the first time, thyme (*Thymus vulgaris* L.*)* against enteral and encysted (parenteral) phases of *Trichinella spiralis* in mice compared with albendazole, and detect their effect on inducible nitric oxide synthase (iNOS) expression. Oral administration of 500 mg/kg of myrrh and thyme led to adult reduction (90.9%, 79.4%), while 1,000 mg/kg led to larvae reduction (79.6%, 71.3%), respectively. Administration of 50 mg/kg of albendazole resulted in adult and larvae reduction (94.2%, 90.9%). Positive immunostaining of inflammatory cells infiltrating intestinal mucosa and submucosa of all treated groups was detected. Myrrh-treated mice showed the highest iNOS expression followed by albendazole, then thyme. On the other hand, both myrrh and thyme-treated groups showed stronger iNOS expression of inflammatory cells infiltrating and surrounding encapsulated *T. spiralis* larvae than albendazole treated group. In conclusion, myrrh and thyme extracts are highly effective against both phases of *T. spiralis* and showed strong iNOS expressions, especially myrrh which could be a promising alternative drug. This experiment provides a basis for further exploration of this plant by isolation and retesting the active principles of both extracts against different stages of *T. spiralis.*

Trichinellosis is a world-wide important parasitic zoonosis with global distribution. The different cultural eating habits represent the main factor of human infections which mainly occur through ingestion of infective larvae in undercooked pork products (Pozio 2007).

All stages of development of *Trichinella spiralis*, adult, migratory, and encysted stages are found in the same host and it infects a wide variety of mammalian hosts, so this parasite has been commonly used as an experimental model to estimate the effect of many anthelmintic agents (Yadav & Temjenmongla 2006).

Albendazole and mebendazole are the principal anthelmintic drugs for the treatment of trichinellosis (Gottstein et al. 2009). However, they have limited bioavailability, a high degree of resistance, and weak activity against encapsulated larvae (Caner et al. 2008). Furthermore, some of these drugs are contraindicated in pregnancy and children under three years (Yadav & Temjenmongla 2012), while others are thought to be carcinogenic (Shalaby et al. 2010). So, there is an intense need for safe and effective antitrichinellosis drugs, especially those from natural agents (Yadav & Temjenmongla 2012), as they have less toxicity and are free from adverse effects (Basyoni & El-Sabaa 2013).

Myrrh is derived from the stem of different *Commiphora molmol* and contains volatile oil, resin, and gum. It is one of the most widely used plants in traditional medicine as an effective analgesic, antipyretic, antibacterial, and antifungal (Dolara et al. 2000). It has fasciolocidal, schistosomicidal, insecticidal, molluscicidal activities, and a potent activity against both intestinal and muscular stages of *T. spiralis*(Basyoni & El-Sabaa 2013).

Thyme (*Thymus vulgaris*) is one of the popular culinary herbs in southern Europe and Mediterranean regions. Thymol is a natural compound isolated from thyme. It has antiseptic, antifungal, and strong antibacterial activities (Dorman & Deans 2000, Mathela et al. 2010). It also has a powerful antioxidant activity (Ündeğer et al. 2009, Ahmad et al. 2010, Zarrini et al. 2010). Thyme was found to have a significant antiparasitic effect against bird protozoan*, Trichomonas gallinae* (Nasrabadi et al. 2012), and in vitro scolicidal effects (Moazeni et al*.* 2012).

Nitric oxide (NO) is produced from arginine by inducible NO synthase (iNOS) from activated T-cells. It is one of the secretory products of macrophages which contribute to the host defense function. It kills or at least suppresses many parasites,*Trypanosoma cruzi*, *Plasmodium*, *Leishmania major*, *Schistosoma*, and *Toxoplasma gondii*(Ascenzi & Gradoni 2002, Boczon’ et al. 2004). During the intestinal phase of*T. spiralis* infection, NO plays a minor role in the expulsion of*T. spiralis* adults; however, it essentially participates in intestinal pathology (Boczon’ et al. 2004) *.* During the muscular phase many structural and biochemical changes occur in muscles which are regulated by oxygen and nitrogen free radicals generated by both the host and the parasite. These free radicals can combine with NO to form peroxynitrate, which leads to injury or death of the muscle fibres. This induced iNOS activity in the skeletal muscle of *T. spiralis*-infected mice may participate in the host’s biochemical defense mechanism (Boczon’ & Wargin 2000, Boczon’ et al. 2002, 2004), 2004).

The present study aimed at investigating the effect of myrrh and thyme extracts on both the intestinal and muscular phases of *T. spiralis* infection in mice in comparison with albendazole (reference drug). It also aimed at studying the iNOS expression following treatment by each drug during both phases of infection and discussing its relevance to the pathology of the parasite and defense mechanisms of the infected host.

## MATERIALS AND METHODS

*Experimental animals and model -* One hundred and thirty parasitic free BALB/c mice (25-30 g) aged six-eight weeks were used. They were obtained from the Faculty of Medicine, Assiut University, Assiut, Egypt, and maintained under controlled light and temperature with standard diet and water supplies. One hundred and twenty of them were infected orally with about 300 larvae per mouse while the remaining 10 were kept as the noninfected nontreated control group. The strain of*T. spiralis* used was originally isolated from the diaphragms of infected pigs obtained from El-Bassatine Abattoir, Cairo. It had been routinely maintained in the laboratory of the Faculty of Medicine, Assiut University, by consecutive passage through BALB/c mice following the method described by Gamble (1996). Briefly, the heavily infected diaphragms of the pigs were minced and digested in 1% pepsin-hydrochloride. After overnight incubation at 37°C, larvae were collected using the sedimentation technique, washed in physiological saline (0.85%) several times, and the number of larvae per mL was counted.

*Reference drug -* Albendazole was supplied as suspension, 20 mg/mL, from the Egyptian International Pharmaceutical Industries Co.

*Plant materials and preparation of extracts -* The aerial parts of*T. vulgaris* L. (thyme), family Limiaceae (1,000 g) were collected from the botanical garden of the Faculty of Pharmacy, Assiut University, during June 2014, then identified by Dr Zidan Ibrahim, Professor of Pharmacognosy, Faculty of Pharmacy, Assiut University. It consists of volatile oil (1.2% v/w) with phenol value not less than 0.5% expressed as thymol. The phenols in the isolated oil are determined by reaction with aminopyrazolone and potassium ferricyanide in ammonical solution with subsequent measurement of absorbance at 450 nm (Evans 2002). The plant was shade dried, crushed, sieved, and kept till use. A voucher specimen was deposited in the Botanical Museum, Faculty of Pharmacy, Assiut University. A 500 g of the shade-dried powdered aerial parts of the thyme was extracted with 70% ethanol by maceration and percolation for 24 h. The extraction process was repeated twice. The alcohol extracts of the plant were pooled together and evaporated under reduced pressure at 45ºC till they were free from the solvent. The solvent-free residue was weighed to give 8.76 g (yield, 17.52%). The dried extract was stored at +4ºC till use. Myrrh is oleo-gum resin (*C. molmol*) family Burseraceae. Its import from Sudan exudes naturally or from incisions made in bark. It contains 7-17% volatile oil, 25-40% resin, 57-61% gum, and 3-4% impurities. It was purchased from the local market in Assiut Governorate, crushed to fine powder and sieved. Ten grams of the plant material were suspended in 100 mL distilled water with the aid of 3% Tween 80 and kept in the refrigerator till use. Chemical tests give yellowish emulsion when triturated with water, and when extracted with 90% alcohol a whitish mass of gum and impurities remains (Evans 2002).

*Cytotoxicity assays (CTAs) on tissue culture cells -* CTAs were performed on mouse fibroblast cell BALB/c 3T3 (VACSERA, Egypt) supplemented with 10% bovine calf serum, 4 mM L-glutamine, 100 IU penicillin, and 100 µg/mL streptomycin (Bioanalyse, Turkey) using the neutral red uptake assay (Repetto et al. 2008).

*Evaluation of microbial contamination and endotoxin production -*Total aerobic microbial count and total combined yeasts/moulds count were used for quantitative enumeration of mesophilic bacteria or fungi that may grow under aerobic conditions in our herbal products using the pour plating technique (EDQM Council of Europe 2014).

The bacterial endotoxin test was performed by the limulus amoebocyte lysate assay (gel-clot technique) as reported (Hussaini & Hassanali 1987).

*Study design groups of animals -* Infected mice were classified into four main groups; A, B, C, and D. Each group had 30 mice. Group A was infected and left untreated as a control group while groups B, C, and D received oral doses of albendazole, thyme, and myrrh, respectively.

*Dose schedule -* Each main group of the treated animals was subdivided into three subgroups (I, II, and III), each subgroup having 10 mice. Animals of subgroup I of group B, C, and D received 50 mg/kg albendazole, 500 mg/kg thyme, and 500 mg/kg myrrh, respectively, starting from the third day post-infection (dpi) for three successive days (Shoheib et al. 2006). They were sacrificed on the seventh dpi to evaluate the effects of the drugs against adult worms (intestinal phase). The numbers of adult worms in the gut were isolated and counted (Denham & Martinez 1970). Animals of subgroups II and III of group B, C, and D received 50 mg/kg albendazole, 1,000 mg/kg thyme, and 1,000 mg/kg myrrh, respectively, starting from the 31st dpi for seven successive days. Mice from subgroup II were sacrificed on the 49th dpi (Yadav & Temjenmongla 2012) to evaluate the effects of the drugs against the larvae and count them (Goettsch et al. 1994). Mice from subgroup III were sacrificed on the 60th dpi (Caner et al. 2008). The infected, nontreated control group (group A) was subdivided into five subgroups (6 mice each). Each subgroup was sacrificed on the seventh, 21st, 30th, 49th, and 60th dpi, respectively. For histopathological and immunohistochemical evaluation, intestinal specimens were taken from sacrificed mice on the seventh dpi, while muscular specimens were taken from sacrificed mice on the 21st, 30th, 49th, and 60th dpi, respectively.

*Histopathological examination -* Intestinal specimens (1 cm from the small intestine at the junction of the proximal 1/3 and distal 2/3) (Nassef et al. 2010) were taken from mice sacrificed on the seventh dpi, while skeletal muscle specimens from the hind legs were taken from mice sacrificed on the 21st, 30th, 49th, and 60 dpi (Zeromski et al. 2005, Monib et al. 2010). These specimens were fixed in 10% formaline, dehydrated, cleared, and then embedded in paraffin blocks. Paraffin sections were taken of 5 µm thickness and stained by haematoxylin and eosin and examined microscopically (Shalaby et al. 2010).

*Evaluation of iNOS expression during the course of infection -* The presence of iNOS protein was analysed by immunohistochemical staining using the avidin biotin immunoperoxidase complex technique (Ultravision Plus Detection System antipolyvalent HRP/DAB, ready to use; Thermo Scientific Corporation, USA). Immunohistochemistry was performed according to the manufacturing protocol. Tissue sections (4-µm thick) of the previously formalin-fixed, paraffin-embedded specimens were cut and mounted. The sections were de-paraffinised and rehydrated in graded alcohol, and endogenous peroxidase was blocked by the use of 3% hydrogen peroxide in methanol for 5 min. For antigen retrieval the slides were immersed in a citrate puffer and put in the microwave for 8 min. The samples were then incubated for 1 h at room temperature with iNOS (Rabbit Polyclonal Antibody; Thermo Scientific Corporation) at a dilution of 1:100. After the application of a secondary antibody, slides were developed using 3-3′-diaminobenzidine chromogen and counterstained with haematoxylin. Negative control slides were made by omitting the primary antibody. Sections from the lung were stained as a positive control.

*Evaluation of immunohistochemistry -* Cytoplasmic NO expression was evaluated by counting positive cells in 10 high power fields in well-labelled areas as determined at low magnification (the most densely labelled areas) and expressed in percentage.

*Statistical analysis -* The collected data were analysed by Statistical Package for Social Sciences v.20 for Windows. All values were expressed as mean ± standard deviation. The significance of differences between the groups was calculated using the ANOVA test. The percentage of reduction was calculated between the treated groups and the control groups. The significance of differences between the groups was calculated using Student’s *t* test. p-value of < 0.05 was considered statistically significant.

*Ethics -* The experimental animal studies were conducted in accordance with the international valid guidelines and were maintained under convenient conditions at the Animal House, Faculty of Medicine, Assiut University.

## RESULTS

*The CTAs -* The optical density (OD)_540_ of each test article was compared with the mean value OD_540_ for the negative control. Our herbal extracts fulfilled the mentioned acceptance criteria. Cell viabilities were > 70% relative to the negative control for thyme and myrrh at their highest concentrations.

*Effects of albendazole, thyme, and myrrh against adult worms -* A significant decrease in the mean number of adult worms was obtained in all treated groups (p < 0.01). The least count was found in group B which received albendazole with efficacy of 94.2% followed by group D which received myrrh with efficacy of 90.3%. The least reduction in the number of adult worms was observed in thyme-treated group (group C) with efficacy of 79.4% (Table).

**TABLE:** Efficacy (as percentage reduction) of albendazole, thyme, and myrrh against adult and encysted stages of *Trichinella spiralis*

Studied groups	Intestinal worm	Count/mouse	Muscle larval	Count/mouse

	Mean ± SD (range)	Efficacy (% reduction)	Mean ± SD (range)	Efficacy (% reduction)
A (infected, nontreated)	31.0 ± 1.6 (29-33)	0	200 7.9 (190-210)	0
B (albendazole)	1.8 ± 0.84^*a*^(1-3)	94.2	18.2 ± 2.9^*a*^(15-22)	90.9
C (thyme)	6.4 ± 1.5^*a*^ (4-8)	79.4	57.4 ± 4.9^*a*^(50-62)	71.3
D (myrrh)	3 ± 1.6^*a*^ (1-5)	90.3	40.8 ± 1.9^*a*^(39-44)	79.6

*a*: statistically significant at p < 0.01; SD: standard deviation.

*Effects of albendazole, thyme, and myrrh against encysted larvae -* A significant decrease in the mean larval count was detected in all treated groups (p < 0.01). The best reduction of larval count was found in group B which received albendazole with efficacy of 90.9%, followed by group D which received myrrh with efficacy of 79.6%. The least reduction was detected in group C which received thyme with efficacy of 71.3% (Table).

*Histopathological effects -* No histopathological changes were observed in both the intestinal and muscular sections of the noninfected, nontreated control group.

Intestinal sections of group A (infected nontreated) on the seventh dpi revealed the presence of adult worm sections within the mucosa together with chronic inflammatory cells infiltrating the mucosa and submucosa. Intestinal sections of treated groups, compared with group A, revealed a marked decrease in inflammatory infiltrate in group B while mild to moderate cellular infiltration was observed in groups C and D.

**Fig. 1:**
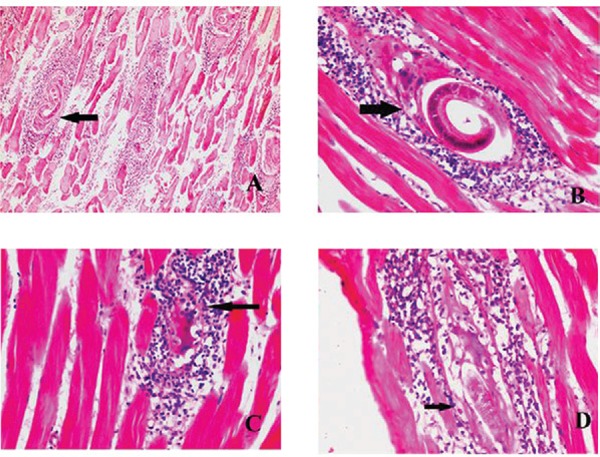
: skeletal muscle sections of *Trichinella spiralis*infected mice stained by haematoxylin and eosin. A: day 30 of infected nontreated group showing multiple encysted larvae surrounded by intense inflammatory cellular infiltrate (200X); B: thyme treated group (1,000 mg/kg) at day 49 showing encysted larvae surrounded by thick intact capsule and intense inflammatory cellular infiltrate (400X); C: albendazole treated groups (50 mg/kg) at day 49 showing homogenised larvae, vacuolation and splitting of the capsule into thin layers (arrow) with diffuse inflammatory cellular infiltration surrounding and invading the capsule (arrow) (400X); D: myrrh treated group (1,000 mg/kg) at day 49 post-infection showing homogenised larvae with broken down incomplete capsule which is completely invaded and surrounded by inflammatory cells (400X).

Muscular sections on the 21st, 30th, 49th, and 60th dpi of group A (infected, nontreated) revealed the presence of a massive number of encysted *T. spiralis* larvae diffusely present in muscles sarcoplasm (Fig. 1A) and a massive number of chronic inflammatory cells in the form of lymphocytes, plasma cells, eosinophils, and histiocytes infiltrating muscle bundles and surrounding the encysted larvae.

On the 49th and 60th dpi, pathological changes in the muscular sections of group C which received thyme showed a fewer number of encysted larvae than the infected, nontreated group with heavier inflammatory cellular infiltration surrounding them. The capsule in most of the larvae appeared thick and complete (Fig. 1B), while in groups B and D which received albendazole and myrrh, respectively, the muscular sections showed much fewer numbers of encysted larvae, most of them showed degenerative changes, areas of thinning and splitting of the capsule into thin layers, areas of breakdown, vacuolization and invasion by inflammatory cellular infiltrate. These changes were more evident in group D (Fig. 1C, D).

*Expression of iNOS producing cells -* Cytoplasmic iNOS expression was detected in chronic inflammatory cells (lymphocytes, plasma cells, and macrophages). No positive iNOS expression could be seen in the intestine or the muscle of the noninfected nontreated control group.

On day 7, iNOS expression was evident in the inflammatory cells infiltrating the mucosa and submucosa of the intestine. In comparison with group A (infected, nontreated), the highest expression was detected in group D (myrrh) followed by group B (albendazole) while group C (thyme) showed expression nearly equal to group A.

**Fig. 2:**
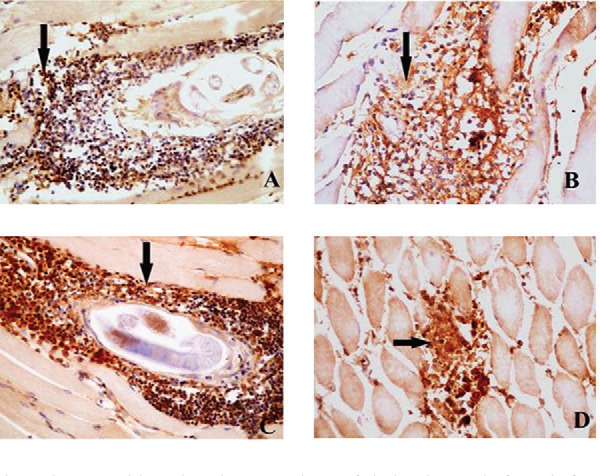
: immunohistochemistry sections of skeletal muscle from infected mice using antiinducible NO synthase (iNOS) antibody. A: infected nontreated group at day 30 showing positive immunostaining of the cellular infiltrates surrounding encysted larvae (400X); B: albendazole treated groups (50 mg/kg) at day 49 showing strong immunostained inflammatory cells surrounding encysted larvae (400X); C: thyme treated group (1,000 mg/kg) at day 49 showing very strong iNOS expression of the cellular infiltrates surrounding the larvae (400X); D: myrrh treated group (1,000 mg/kg) at day 60 showing strong immunostained inflammatory cells infiltrating the muscle bundles (400X).

During the muscular phase, iNOS expression in group A (infected, nontreated mice) was distinct from the 21th up to the 60th dpi in almost all infiltrating cells between muscle bundles and surrounding the encapsulated larvae (Fig. 2A). The highest iNOS expression was detected on the 21st and 60th dpi.

At the 49th and 60th dpi, high iNOS expression was detected in all treated groups including group B (albendazole) (Fig. 2B) in comparison with group A. This effect was more evident in group C (thyme) on day 49 (Fig. 2C), and in group D (myrrh) on the 60th dpi (Fig. 2D).

## DISCUSSION

Some recent studies suggest that there could be a good extent in finding some alternative drug compounds from medicinal plants for an effective management of*T. spiralis* infection (Abu El Ezz 2005, Caner et al. 2008, Shalaby et al. 2010). The present study assessed the effectiveness of myrrh and thyme extracts against different stages of*T. spiralis* in mice compared with the common commercial drug albendazole. The microbial limit of our herbal extracts was within an acceptable range that does not pose health hazards to intended groups or diminish herbal stability (Moreira et al. 2014).

The present results showed a significant decrease in the mean number of adults and larvae in all treated groups. Albendazole showed the highest efficacy against both the intestinal and muscular stages followed by myrrh, while thyme had the least efficacy.

In the present study, albendazole-treated mice were given 50 mg/kg for three successive days starting from the third dpi, which revealed a significant reduction (94.2%) in the numbers of adult worms. Similar efficacies of albendazole and mebendazole, which is another benzimidazole derivative, were reported by previous studies (Chung et al. 2001, Siriyasatien et al. 2003, Yadav & Temjenmongla 2006, Shalaby et al. 2010). Also a significant reduction (efficacy of 90.9%) was revealed when albendazole-treated mice were given the same dose for seven successive days starting from the 31st dpi. Similar efficacy of mebendazole was reported (Yadav & Temjenmongla 2006). However, much lower efficacies of albendazole against encysted muscle larvae were reported by previous studies (Chung et al. 2001, Siriyasatien et al. 2003,Shoheib et al. 2006, Shalaby et al. 2010). The differences in the efficacy of albendazole against both the intestinal and muscular stages depend on the time, dose, and duration of treatment (Siriyasatien et al. 2003).

A dose of 500 mg/kg myrrh given for three successive days (intestinal phase) and 1,000 mg/kg for seven successive days (muscular phase) produced efficacy of 90.3% against adult worms, which was almost comparable with that of albendazole (94.2%), while it decreased to 79.6% against muscle larvae. To our knowledge, only one publication that proved the effectiveness of myrrh against *T. spiralis* has been reported (Basyoni & El-Sabaa 2013). The differences in results might be due to the different doses and schedules.

Besides the potent insecticidal and molluscicidal activities of myrrh (Basyoni & El-Sabaa 2013), it proved efficacious in the treatment of patients with chronic fascioliasis with no observed adverse effects or toxicity (Massoud et al. 2001). It induced a cure rate of 98.09% in schistosomiasis patients with transient side effects (Sheir et al. 2001).

In the present study, thyme efficacy was 79.4% against adult worms (dose of 500 mg/kg) while its efficacy was 71.3% for encysted larvae (dose of 1,000 mg/kg). To our knowledge, no available published literature on thyme (*T. vulgaris)* investigates its anti-*Trichinella* activity, but it has potent scolicidal effects (Moazeni et al. 2012), and showed different significant antiparasitic effects (Mikus et al. 2000, Santoro et al. 2007, Nasrabadiet al. 2012). In general, the efficacy of both plant extracts against encysted larvae was relatively low compared with the adult stage. This may be accounted for by the fact that once the *Trichinella* larvae have become encysted in muscle tissues, therapeutic intervention is generally less feasible as the susceptibility of the cyst to chemotherapeutic agents diminishes with the duration of infection (Yadav & Temjenmongla 2006, 2012).

There are many studies which have investigated the effect of different plant extracts against *T. spiralis* in experimental animals (Abu El Ezz 2005, Yadav & Temjenmongla 2006, 2012, Caner et al. 2008, Shalaby et al. 2010). The efficacies of both plant extracts used in this study were higher than those of previous studies. This may be attributed to the use of high doses and early administration of the extracts during both the intestinal and muscular phases for many successive days.

In this study, histopathological examination of group C (thyme) was similar to that of group A (infected, nontreated) while the mice of groups B and D which received albendazole and myrrh, respectively, showed degeneration of most encysted larvae with splitting, vacuolation, and damage to their capsule combined with diffuse cellular infiltration.

In the present study, iNOS expression on the seventh dpi showed positive inflammatory cells infiltrating the mucosa and submucosa of the intestine of all infected groups. Group D (myrrh) showed the highest expression followed by group B (albendazole) while group C (thyme) and group A (infected, nontreated) showed nearly the same expression. These iNOS expressions, together with the results of drug efficacy for adult expulsion from the intestine, reinforce previous findings which indicated that NO synthesised by iNOS is not essential for the expulsion of *T. spiralis* adults from mice; however, they play a crucial role in the induction of the T-helper 2-mediated severe enteropathy that accompanies the intestinal phase (Lawrence et al. 2000, Abd El Aal et al. 2003, Boczon’ et al. 2004).

The results of the present study showed positive iNOS expression in nearly all cells infiltrating infected muscles from the 21st up to 60th dpi in group A (infected, nontreated). The highest expression was on the 21st and 60th dpi. This result confirms the idea of the increase of iNOS expression in the muscles on day 21 and additionally on 70th dpi (Boczon’ & Wargin 2000, Boczon’ et al. 2004). Consistent with Zeromski et al. (2005), strong iNOS expression could be seen in the inflammatory cells infiltrating and surrounding encapsulated *T. spiralis* larvae in infected muscles after albendazole treatment. These results confirm previous biochemical data which reported that albendazole treatment during experimental trichinellosis resulted in the stimulation of iNOS expression. Both thyme and myrrh-treated groups (groups C and D) showed even stronger iNOS expression which may be due to their reported powerful antioxidant activities (Ündeğer et al. 2009, Ahmad et al. 2010, Zarrini et al. 2010, Fathy 2011). Also the inflammatory cells surrounding the encysted larvae produce high levels of reactive oxygen species, including NO and other free radicals (Bruschi & Lucchi 2001).

Consistent with Boczon’ et al. (2004), Kołodziej-Sobocińska et al. (2007), and Issa et al. (2008), iNOS expression in the present study lasted until the end of the study (60th dpi), which may indicate the protective role of iNOS against early infection (intestinal phase) and encysting larvae (Issa et al. 2008). Expression of iNOS and its impact on the pathology of infected tissue have been found in some other tissue parasites (Jarillo-Luna et al. 2002,Clark et al. 2003, Long et al. 2004).

The results of the present study showed that myrrh and thyme extracts were both effective against both the intestinal and muscular stages of *T. spiralis*, especially myrrh with results nearly comparable to those achieved by albendazole. Both myrrh and thyme extracts showed higher iNOS expression in comparison with albendazole which may effectively modulate the pathological response, immune and defense mechanisms of the infected host. The active principles of both extracts should be isolated and retested against different stages of*T. spiralis.*
